# On Reducing Dislocations

**Published:** 1810-12

**Authors:** Brudford Wilmer

**Affiliations:** Coventry


### *To the Editors of the Medical and Physical Journal.*

### *On reducing Dislocations.*

GENTLEMEN,

THE following remarks on dislocation of the limbs have occurred, on reading a very interesting paper on the subject, published in the Medical and Physical Journal in April, 1808. After offering some very sensible strictures on the present mode of reducing luxations, in counteracting, by violent extension, the resistance of the muscles, (as he emphatically terms it) *by main force of surgery;* Mr. Carwardine proposes to apply a regular and long continued extension, to exhaust their action. This he illustrates by the following case.

A strong muscular man dislocated his shoulder. Attempts to reduce it were ineffectually made in the country where the accident happened; and after an interval of fourteen days, he applied to an hospital which Mr. Carwardine was then attending.

One of the senior surgeons proceeded to the reduction of the joint. After repeated and violent attempts, which proved ineffectual, it was proposed to the man to remain in the hospital till the following day, and in the interim, that he should lose blood, take a cathartic, and keep a relaxing poultice on the joint. His business, however, not allowing of a longer continuance in the hospital, he was about to quit it, when Mr. Carwardine persuaded him to go to his lodgings. Having provided the necessary apparatus, the patient was placed on a stool, the scapula and trunk firmly fixed, and a set of pullies accurately adjusted and secured to the condyles of the humerus, for the purpose of making extension. The cord was now tightened with a force just sufficient to counteract the gravity of the limb, and prevent its falling to his side. Mr. W—— with one hand held the wrist, to preserve the fore arm bent, and with the other held the cord to keep it from relaxing, but without tightening it. This extension was continued for ten minutes, without producing any manifest effect. As the extension gave very little pain, it was slightly increased. In five minutes more the man grew very restless, would not allow that he then suffered pain, but said, *he felt very faint*, begged that Mr. C. would desist, and asked for water, which was refused, under the idea, that if he fainted the muscles would be completely relaxed. At this moment he changed countenance, and the Deltoid muscle was perceived to be tremulous. The cord was tightened a little: the arm followed it; the cord being now relaxed, the head of the bone slipped into its place, and the reduction was accomplished without any perceptible sound.

Mr. Carwardine attributes the success of this experiment to the long continued extension, having exhausted the action of those muscles connected with the dislocated limb; but from cases which have occurred in my practice, it seems more probable, it was the effect of that disposition to faint, which the man complained of towards the latter end of the experiment. In a state approaching to syncope, the circulation becomes weak, the action of all the voluntary muscles is nearly suspended, and if at that favourable moment, an extension could be made in cases of dislocated bones, I believe it would be generally successful. Surgeons who have seen much of practice must have remarked, when they have been called to patients who have suffered these accidents in a state of intoxication, with how much comparative ease, the reduction has been accomplished. In this state all the powers of the body are enfeebled, and the muscular action, for a time, almost destroyed.*

Previous to the action of an emetic, the face becomes pale, and faintness is generally induced. At this critical time, if an extension is made, the reduction of luxated bones is accomplished with a facility, that will surprise those who have not seen the experiment. When cases have occurred in my practice, which have resisted the common methods of reduction, I have ordered an antimonial emetic, and making an extension when the patient first complains of faintness or sickness, have always succeeded, and with very little exertion of force.

Mr. Lloyd, aged about forty, a very strong and muscular man, dislocated his shoulder by a fall down a precipice; as it was late at night, he got no assistance till the following day. At seven o'clock in the morning, an eminent surgeon of this city, was called to him. The trunk of the body was firmly fixed to a wall, and extension made from the lower part of the humerus, with the fore-arm bent, without success. Pullies were then applied, and much force used, but the head of the bone was immoveably fixt. Extension was then made in every direction in vain. These experiments were reiterated during the space of an hour, till the patient and the surgeon were both much exhausted. At eight o'clock, I was called into consultation, and proposed,

* Mr. Chesher has published a case, in which the reduction of a luxated Os Humeri was facilitated by the exhibition of emetic Tartar. Med. Journal, Vol. 8, p. 189.

that five grains of tartarised antimony should be given, every thing being prepared for extension; in five minutes the patient said he felt ill, in three more that he thought he should be sick. By a moderate degree of force applied to the lower part of the bone, in the same direction as that first applied; in a few seconds, the reduction was accomplished with very great ease.

On the fourth of September last, a stout muscular young man of this city was thrown out of an open carriage and dislocated his arm. The next day I was desired to assist his surgeon, Mr. Kann, in the reduction. He had a very bad night, without sleep, in constant pain, was feverish, and the parts over the joint, tense and swelled.

A very forcible extension was made in the usual way, but the head of the bone was firmly fixt under the pectoral muscle. The patient was in a state, in every respect, the most improper, for the application of much force; I, therefore, proposed to Mr. Kann, to give him five grains of tartarised antimony. In seven or eight minutes he complained of being faint. I now remarked to Mr. Kann, the alteration which had taken place in the state of the parts about the joint. The muscles, before hard and tense, had become soft and flabby. Extension being then made in the same direction and manner as before, the reduction was immediately accomplished.

It was the opinion of Dr. Hunter, that in complete luxations of the os humeri, the capsular ligament was always ruptured, and that the nature of the laceration would sometimes render the reduction impracticable. And Mr. Thompson* has supposed, that the head of the bone might be tightly embraced by the tendons of the subscapularis and teres minor muscles passing obliquely over it, and thus form an impediment to reduction. I believe it is now generally admitted, when these difficulties occur, that they are occasioned by the muscular power counteracting, with effect the extending force applied to them. Mr. Pott, aware of this circumstance, has given precise directions (by the position of the limb) to place the muscles, surrounding and connected with the luxated bone, in that state where they are likely to give the least resistance. But it will be found impossible to place a limb in any position, in which some of the muscles will not be more or less elongated. If the flexor muscles are relaxed, the extensors are, in the same proportion, stretched. Hence, it

* Medical Observations and Enquiries, Vol. 2, p. 355. Potts Works, Fol. Edit. p. 693.

appears to be a great desideratum to affect the system in such a manner that *all the muscles* connected with the luxated bone may for a short time be deprived of their powers of making resistance.

In cases where great force and violent extension have produced no effects upon the dislocation, it has been the practice of surgeons to reduce the strength of the patient by large bleeding, baths, cathartics, and to relax the parts by fomentations, poultices, &c. That these sometimes fail is too well known to be insisted upon. The exhibition of an antimonial emetic in a few minutes reduces the muscles into a state of inaction; the other plan will not have the same effect in as many days. From so much evacuation constitutional effects may arise. From the former plan the patient's general health may be improved, and his recovery will be very speedy. He should go to his bed after the operation of the emetic, when a perspiration ensues, which will remove that feverish action of the system, the general accompaniment of accidents of this nature. The muscles also at the time of the reduction are so much relaxed that no pain attends the operation.

I am, &c.

BRUDFORD WILMER.

*Coventry, Oct.* 15, 1810.

---

### *To the Editors of the Medical and Physical Journal.*

### *Society for the Relief of Widows and Orphans of Medical Men.*

GENTLEMEN,

